# Polyploidy-associated autophagy promotes larval tracheal histolysis at *Drosophila* metamorphosis

**DOI:** 10.1080/15548627.2023.2231828

**Published:** 2023-07-09

**Authors:** Beatriz Pino-Jiménez, Panagiotis Giannios, Jordi Casanova

**Affiliations:** a Department of Cell and Tissues, Institut de Biologia Molecular de Barcelona (CSIC), Barcelona, Catalonia, Spain; b Institut de Recerca Biomèdica de Barcelona, (IRB Barcelona), The Barcelona Institute of Science and Technology (BIST), Barcelona, Catalonia, Spain

**Keywords:** Apoptosis, autophagy, *Drosophila*, polyploidy, progenitor, trachea

## Abstract

Polyploidy is an extended phenomenon in biology. However, its physiological significance and whether it defines specific cell behaviors is not well understood. Here we study its connection to macroautophagy/autophagy, using the larval respiratory system of *Drosophila* as a model. This system comprises cells with the same function yet with notably different ploidy status, namely diploid progenitors and their polyploid larval counterparts, the latter destined to die during metamorphosis. We identified an association between polyploidy and autophagy and found that higher endoreplication status correlates with elevated autophagy. Finally, we report that tissue histolysis in the trachea during *Drosophila* metamorphosis is mediated by autophagy, which triggers the apoptosis of polyploid cells.

**Abbreviations:** APF: after pupa formation; *Atg*: autophagy related; *btl*: breathless; *CycE*: Cyclin E; DT: dorsal trunk; *fzr*: fizzy-related; L3: larval stage 3; PBS: phosphate-buffered saline; RI: RNAi; Tr: tracheal metamere; *yki*: yorkie.

## Introduction

Animal development does not occur only through the building of new tissues and organs but also through death decisions that result in the breakdown of existing structures and contribute to the plastic remodeling of tissue architecture. Indeed, these events are not limited to development but also occur to maintain the homeostasis of the adult organism. Numerous examples of this phenomenon are observed in lower organisms, through well-studied procedures of programmed cell death through to humans, where structures like the Wolffian or Müllerian ducts undergo regression during the development of fetal gonads and the endometrial tissue architecture is modified in response to hormonal cues [1–3]. In some cases, the disassembly of an organ, and thus the disposal of its given purpose, is associated with the generation of a new organ that will retake the eliminated functions or acquire a new essential role during the next developmental stage. In this instance, new organs develop from cells that retain progenitor potential. A prototypic case of such events is the metamorphosis of *Drosophila*, a holometabolous insect, a process driven by the molting hormone ecdysone. During *Drosophila* metamorphosis most larval cells die and only some survive – the adult progenitor cells – to give rise to the adult organism (see for instance [4,5]). However, to date, we still lack a clear picture of the events responsible for the death of most larval cells and, at the same time, for the proliferation and differentiation of the adult progenitor cells into new adult organs.

**Figure 1.**
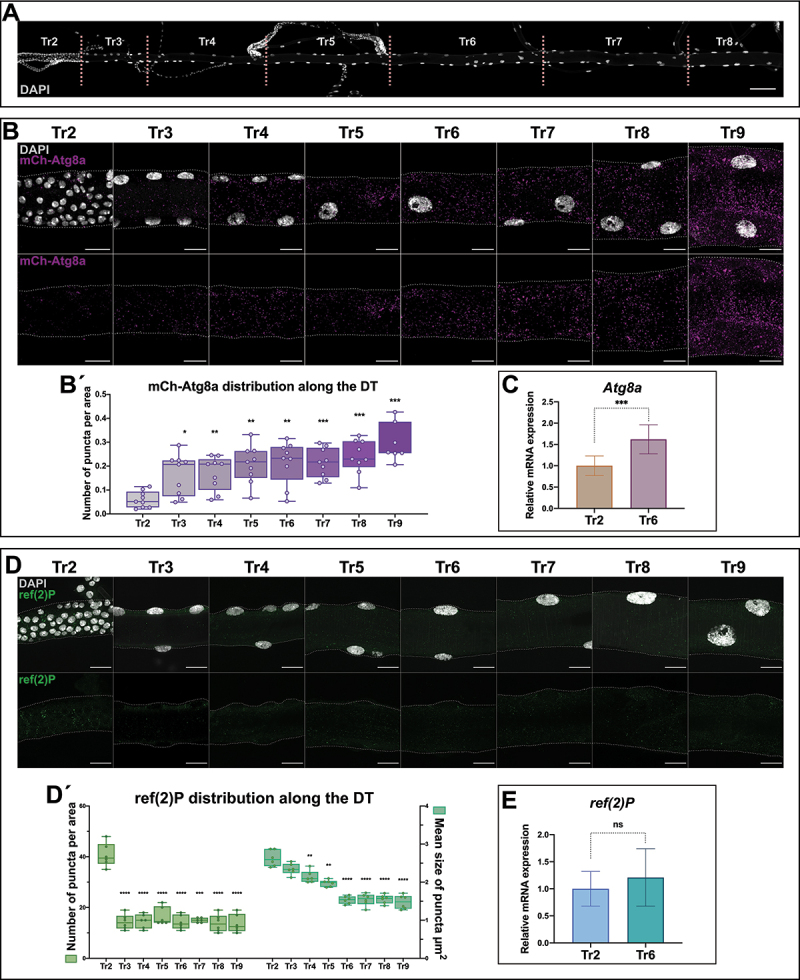
Differential autophagy along the dorsal trunk of the larval trachea. (A) the DT is a tubular structure divided into 10 metameres (Tr1-Tr10). Tr2 to Tr8 metameres are shown here, dissected from a larva of the L3 stage. Cell nuclei are labeled with DAPI. Cells of Tr2 are the diploid adult progenitors, while the cells of the rest of the metameres are large polyploid larval cells. Other minor branches that form the larval tracheal system are connected to the dorsal trunk. Scale bar: 100 μm. (B) Magnification of metameres Tr2-Tr9 from L3 larvae carrying the mCh-Atg8a reporter, in magenta; cell nuclei are labeled with DAPI, in white. Scale bars: 20 μm. (B´) Box plots show the distribution of the values of mCh-Atg8a puncta per area in each metamere, which show significant differences between cells of Tr2 and those of the rest of the metameres. For the analysis of variance between groups, Welch’s ANOVA (*p* = 0.0001) was applied followed by Dunnett’s T3 multiple group comparison test. (alpha set at 0.05. *p < 0.05, **p < 0.01, ***p < 0.0005). *n* = 9 individuals per group. Error bars represent SD of means. (C) Relative expression levels for the mRNAs of Atg8a in Tr2 and Tr6 metameres, the transcripts of the gene found enriched in Tr6 cells. (***p < 0.0001, Unpaired t-test). *n* = 6 control btlGal4/+ individuals. (D) Magnification of metameres Tr2-Tr9 of L3 larvae. ref(2)P in green and cell nuclei are labeled with DAPI, in white. Scale bars: 20 μm. (D´) Box plots show the distribution of the values of ref(2)P dots per area and their average size in each metamere of the DT (Tr2-Tr9). The number of ref(2)P dots is higher in Tr2 in comparison to polyploid metameres, and their size decrease progressively along the DT. For the analysis of variance between groups, in both cases, Welch’s ANOVA (*p* = 0.0001) was applied followed by Dunnett’s T3 multiple group comparison test. (alpha set at 0.05. **p < 0.005, ***p < 0.0005, ****p < 0.0001). Only statistically significant differences are shown. *n* = 6 per group. btlGal4/+ individuals were used. Error bars represent SD of means. (E) Relative expression levels for the mRNAs of ref(2)P in Tr2 and Tr6 metameres; the transcripts of the gene do not show detectable differences of expression between metameres. (*p* = 0.33, Unpaired t-test). *n* = 9 control btlGal4/+ individuals.

A major challenge in identifying these events is the difference between the features of the cells destined for destruction and those of progenitor cells, which hinders their comparative analysis. In this regard, a particularly appropriate system to study this question is the larval trachea of *Drosophila*. The *Drosophila* larval respiratory organ consists of two main tracheal branches, the dorsal trunks (DTs), each comprising 10 metameres (Tr1-Tr10). Only one of the later, namely the second of these metameres (Tr2), is composed of presumptive progenitor cells of the adult trachea, while the remaining cells of the DT are thought to die at metamorphosis. Of note, the cells of all metameres, both the progenitors and those to be eliminated during metamorphosis, are fully differentiated and perform their larval tracheal function. However, there is a major difference between these cells: those of Tr2 are diploid while those in the other metameres are polyploid, like most other larval cells (Figure 1A) [6–9].

Using this model, here we focused on what could account for the difference in the behavior of DT cells from Tr2 (proliferation, survival and final differentiation) and those of the other metameres (undergoing programmed cell death) at metamorphosis. In particular, we centered on autophagy, a lysosome-mediated cellular degradation process that can either contribute to survival, allowing cells to recycle their constituents, or to cell death at elevated levels [10,11], and that has an established role in the metamorphosis of *Drosophila*. More precisely, autophagy defines the metamorphosis of structures such as the larval midgut, the fat body, and the salivary glands in this organism [12–15]. However, the specifics of autophagy differ in each of these organs, an observation that is consistent with a tissue-dependent role of this process during metamorphosis (for reviews, see [16–18]).

Here we report an uneven distribution of markers used to monitor autophagy along the DT of the larval trachea, higher in polyploid larval cells that are destined to be removed during metamorphosis than in the diploid progenitors of Tr2. Additionally, we studied the association of autophagy with polyploidy, its role in the metamorphosis of the tracheal system, and its relation with the role of apoptosis in the same process. Our results reveal new features of autophagy regulation and function and provide insight into how the uneven degree of autophagy associated with polyploidy can account for its differential role in cell survival or cell death in a variety of physiological scenarios.

## Results and discussion

### Differential autophagy in the larval trachea

To monitor autophagy along the dorsal trunk (DT) of L3 wandering larvae, we first used a 3×mCherry-Atg8a reporter (from now on referred to as mCh-Atg8a) that labels all autophagic structures [17,19,20]. We detected the mCh-Atg8a protein, present in the form of distinct puncta in the cytoplasm, in all cells of the DT and we quantified the number of puncta per area to identify the pattern of accumulation along the tracheal tube. Of note, in the diploid cells of Tr2, the number of mCh-Atg8a puncta/area was consistently lower in comparison to the polyploid cells of the rest of the metameres (Figure 1B). The difference in Atg8a protein accumulation observed in polyploid versus diploid cells was also consistent in terms of relative *Atg8a* expression, which was enriched in cells of Tr6 in comparison to diploid cells of Tr2 (Figure 1C).

To further assess the evidence of differential autophagic activity between the diploid tracheal progenitor cells of Tr2 and the polyploid larval cells of the same organ, we used an antibody that recognizes the ref(2)P/SQSTM1/p62 protein as an additional marker to monitor the process. ref(2)P is a receptor for ubiquitinated proteins that acts as an autophagic cargo and is therefore subject to autophagic degradation [21,22]. The levels of ref(2)P are inversely correlated with autophagic activity, a property that has been exploited to widely use ref(2)P as a reporter of autophagy in *Drosophila* [17,23,24]. In agreement with the results obtained using the mCh-Atg8a reporter, ref(2)P was detected in a punctate pattern all along the DT with significant differences between the diploid cells of Tr2 and the polyploid cells of the rest of the metameres (both number and average size of the cytoplasmic puncta/area were lower in the latter) (Figure 1D). The difference in ref(2)P accumulation between diploid and polyploid cells is not a consequence of transcriptional regulation as assessed by qPCR of *ref(2)P* transcripts between the Tr2 metamere, with diploid cells, and the Tr6 metamere with polyploid cells (Figure 1E).

### Differential tracheal autophagy depends on tracheal activity of *Atg* genes

We next studied whether autophagy in the trachea depends on the tracheal activity of *Atg* genes. To this end, we examined and quantified the ref(2)P signal in the tracheas of larvae where different *Atg* genes that encode proteins required for autophagosome formation were either absent or knocked down (see Materials and Methods). In brief, in tracheas dissected from *Atg14* mutants, both the size and the number/area of ref(2)P puncta increased along the DT compared to the controls. The changes observed were more prominent in polyploid cells while in Tr2 cells only the size of the puncta showed a significant difference (*p* <*0.05*) in comparison to the control (Fig. S1), thereby suggesting low autophagic activity in cells of this metamere in normal conditions. To confirm the trachea-specific role of the autophagy machinery, we used a *btl*Gal4 driver [25] to trigger the tracheal expression of RNAi constructs targeted to *Atg1*. Also, in this case, both the size and the number/area of the ref(2)P puncta were significantly higher compared to the controls, a change that was observed in all the polyploid metameres of the DT (Fig. S1).

### Autophagy and polyploidy

**Figure 2.**
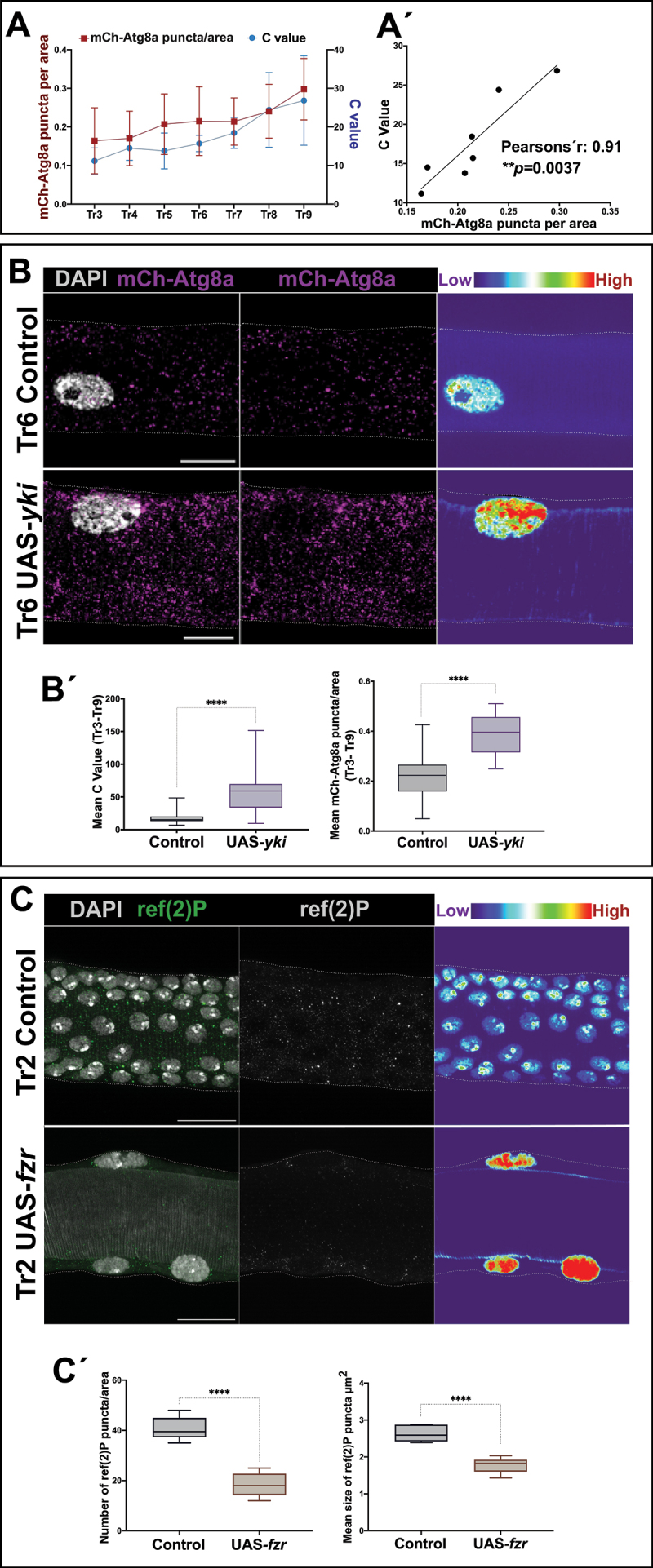
Correlation between autophagy and polyploidy. (A) Mean value of mCh-Atg8a puncta along polyploid metameres (Tr3-Tr9) and mean value for the number of endocycles in the cells of the same metameres. *n* = 3–7 cells per metamere, 9 individuals. Error bars represent SD of means. (A´) Correlation chart of mCh-Atg8a puncta and C value of polyploid cells along the DT. A positive linear correlation is revealed with high mCh-Atg8a levels associated with elevated polyploidy (Pearson’s *r* = 0.91, **p = 0.0037). (B) Ectopic expression of yki results in an increase of endocycles and mCh-Atg8a puncta per area. Magnification of metamere Tr6 of L3 larvae in mCh-Atg8a;btlGal4 (control) and mCh-Atg8a;btlGal4>UAS-yki. mCh-Atg8a in magenta and cell nuclei are labeled with DAPI, in white. Thermal scale represents pixel intensities in sum projections for the DAPI channel. Scale bars: 20 μm. (B´) Box plots for the summary of C values (left chart) and mean values of mCh-Atg8a puncta/area (right chart) in Tr3-Tr9 of control and mCh-Atg8a;btlGal4>UAS-yki (alpha set at 0.05. ****p < 0.0001, Unpaired t-test, Welch’s correction) *n* = 6 individuals per group. Error bars represent SD of means. (C) Switch from diploidy to polyploidy results in increase of autophagy levels as monitored by ref(2)P antibody. Magnification of Tr2 metameres from L3 larvae of btlGal4 (control) and btlGal4>UAS-fzr; ref(2)P in green and cell nuclei are labeled with DAPI, in white. In the second panels, ref(2)P is shown in a single white channel. Thermal scale pseudo color represents pixel intensities in sum projections for the DAPI channel. Scale bars: 20 μm. (C´) Box plots for the number per area (left chart) and the average size (right chart) of ref(2)P puncta in btlGal4 (control) and btlGal4>UAS-fzr (alpha set at 0.05. ****p < 0.0001, two tailed Unpaired t-test, Welch’s correction) *n* = 5 individuals per group. Error bars represent SD of means.

The above observations with either mCh-Atg8a or ref(2)P antibody indicated a difference in the autophagy levels between diploid and polyploid cells of the tracheal DT. In addition, an up-trend in the accumulation of mCh-Atg8a puncta was observed amongst the population of endoreplicating cells of the DT, starting lower in cells of the Tr3 and gradually increasing along the posterior metameres (Figure 2A). We confirmed the significance of this observation by staining with an antibody detecting the endogenous Atg8a, which showed a consistent gradual increase along the DT (Fig. S2). Taking these results in consideration we used either mCh-Atg8a, the Atg8a or the ref(2)P antibodies to evaluate autophagy in different genetic combinations depending on experimental settings.

Quantification of the DNA content (C Value) of DT cells at the L3 stage (see Materials and Methods for details) revealed a key difference in the number of endocycles undergone amongst the polyploid cells of the DT. Also in this case, a gradual up-trend was noted from the anterior Tr3 to the posterior metameres, starting from a low median C value of 11.17 for Tr3, which gradually increased up to 24.39 and 26.84 for Tr8 and Tr9, respectively (Figure 2A). A comparison of these data from the mCh-Atg8a reporter and the DNA content quantifications allowed us to establish a positive correlation between the level of polyploidy and autophagy along the DT (Pearson’s *r* = 0.91, *p=*0.0037) (Figure 2A).

To further assess this correlation, we induced constitutive *yki* activity in all the trachea (see Materials and Methods), which multiples the number of cell cycles and thus gives rise to a Tr2 with many more cells and to the other tracheal metameres with a higher degree of polyploidy in their cells [9]. In this case, as expected, we found a strong increase (*p < 0.0001*) in the C value for the *btl*Gal4>UAS*-yki* larvae, in all the metameres comprised of polyploid cells (mean C value [Tr3-Tr9] = 57.1) compared to the control (mean C value [Tr3-Tr9] = 17.83). This increase in the endoreplication cycles was coupled with a significant increase (*p < 0.0001*) in the mean number of mCh-Atg8a puncta/area detected (mean value of mCh-Atg8a puncta/area [Tr3-Tr9] *btl*Gal4>UAS*-yki* = 0.38, mean value of mCh-Atg8a puncta/area [Tr3-Tr9] Control = 0.215) (Figure 2B). Consistently, a reduction of endocycling by means of a *yki*RNAi construct under the control of a *btl*Gal4 (mean C value *btl*Gal4>*yki*RNAi [Tr3-Tr9] = 9.62 compared to the control mean C value [Tr3-Tr9] = 17.83, *p < 0.0001*) revealed a significant decrease in the number of Atg8a in comparison to the control (0.17 compared to the control 1.67, *p < 0.0001*) (Fig. S3). To further explore the potential correlation between polyploidy and autophagy in our system, we forced expression of a UAS-*CycE* construct using a *btl*Gal4 driver, which resulted in a significant increase (*p* = 0.0002) in polyploidy (mean C value [Tr3-Tr9] = 34.77 compared to the control mean C value [Tr3-Tr9] = 17.83), followed by a concomitant increase in the number of Atg8a puncta/area (2.61 compared to the control 1.67, *p* = 0.03) (Fig. S3).

To confirm that polyploidy was the cause of increased levels of autophagy in the tracheal cells, we resorted to a genetic tool to induce a switch from mitotic divisions to endocycling in Tr2 cells. Given that polyploidy in tracheal metameres depends on the activity of the *fzr* (*fizzy-related*) gene [7,9], we induced *fzr* expression in all cells of the tracheal system using a *btl*Gal4 driver. Upon *fzr* expression, the DT cells from Tr2 entered the endocycle and became polyploid. Under these conditions, using the ref(2)P antibody, we observed that both the average size and the number/area of ref(2)P puncta were significantly reduced when compared to control diploid Tr2 cells (*p < 0.0001* for both comparisons) (Figure 2C). We also examined the effects of diverting polyploid metameres into mitotic divisions by downregulating *fzr* via a *fzr*RNAi construct expressed using a *btl*Gal4 driver. Consistently, we found a reduction in the number of Atg8a puncta/area in the transformed metameres (0.22 compared to the control 1.67, *p < 0.0001*) (Fig. S3). Taken together, our results suggest a causal relationship between diploidy and decreased autophagy levels in Tr2 in wild-type conditions. On the other hand, we observed that other metameres showed an increase in autophagy levels depending on the number of endocycles.

### Autophagy downregulation in the trachea impairs its remodeling at metamorphosis

After we identified the distinct pattern of the autophagy markers along the metameres of the DTs, we sought to determine the specific role of this process in the tracheal system. To this end, we again drove the tracheal expression of RNAi constructs targeting *Atg1* and *Atg8*, as previously described (see Materials and Methods). We were not able to detect severe malformations in the majority of the tracheas of the *btl*Gal4>UAS*-atg1*^*RI*^ and *btl*Gal4> UAS*-atg8*^*RI*^ larvae examined. However, we noticed a decrease in the degree of polyploidy in tracheas upon autophagy inhibition (mean C value [Tr3-Tr9] = 12.49 compared to the control mean C value [Tr3-Tr9] = 17.83, *p* = 0.006) (Fig. S3), consistent with the connection between the autophagy pathway and cell-cycle progression (for a review see [26]).

**Figure 3.**
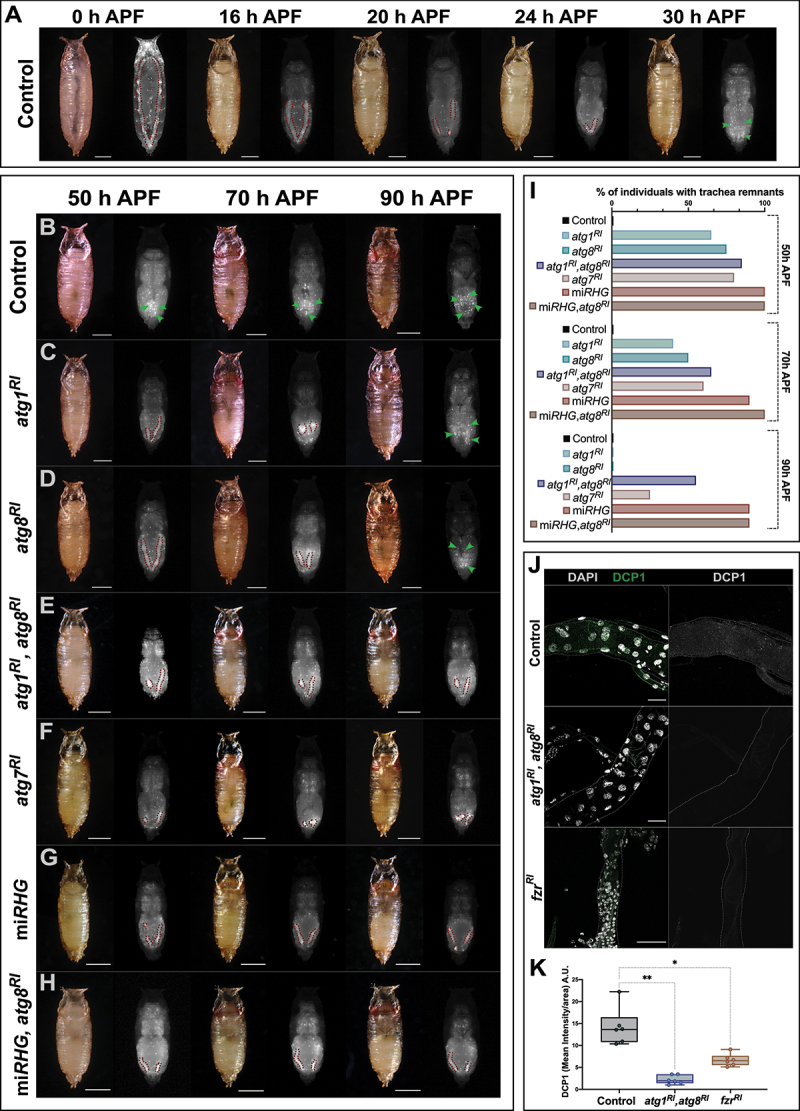
Degradation of the larval DT during metamorphosis is mediated by autophagy eliciting apoptosis. (A) Representative images of control pupae (btlGal4>UAS-DsRed) 0-30 h APF. Larval DT remnants in the abdomen are outlined by a red dashed line. At 30 h APF there are no visible tubular structures and scattered cells, belonging to the newly formed branches of the adult respiratory system can be detected (green arrowheads). (B-H) Representative images of pupae at extreme timepoints showing the delay or defective elimination of DT. Indicative phenotypes of pupae from (B) btlGal4>UAS-DsRed (control), (C) btlGal4>UAS-DsRed,UAS-atg1^RI^, (D) btlGal4>UAS-DsRed,UAS-atg8^RI^, (E) btlGal4>UAS-DsRed,UAS-atg1^RI^,UAS-atg8^RI^, (F) btlGal4>UAS-DsRed,UAS-atg7^RI^, (G) btlGal4>UAS-DsRed,UAS-miRHG and (H) btlGal4>UAS-DsRed,UAS-miRHG,UAS-atg8^RI^. Larval DT remnants noted in the abdomen are outlined by a red dashed line, and scattered cells from branches of the newly formed adult trachea are indicated by green arrowheads. 50 h APF, 70 h APF and 90 h APF are shown. Scale bars: 500 µm. (I) Percentage of individuals found with DT remnants at 50, 70 and 90 h APF. btlGal4>UAS-DsRed (control) *n* = 25, btlGal4>UAS-DsRed,UAS-atg1^RI^ *n* = 24, btlGal4>UAS-DsRed,UAS-atg8^RI^ *n* = 27, btlGal4>UAS-DsRed,UAS-atg1^RI^,UAS-atg8^RI^ *n* = 22, btlGal4>UAS-DsRed,UAS-atg7^RI^ *n* = 25, btlGal4>UAS-DsRed,UAS-miRHG *n* = 36, btlGal4>UAS-DsRed,UAS-miRHG,UAS-atg8^RI^ *n* = 35. (J) Tr6 metamere dissected from pupae at 12 h APF, from btlGal4 (control), btlGal4>UAS-atg1^RI^,UAS-atg8^RI^ and btlGal4>UAS-fzr^RI^. Apoptosis, marked by DCP1 staining was significantly reduced when autophagy was impaired by combined inhibition of Atg1 and Atg8 and when polyploidy was switched into diploidy by fzr^RI^. in left panels, DCP1 is labeled in green and nuclear DNA by DAPI in white. In right panels, DCP1 is shown is single channel in white. Scale bars: 50 µm. (K) Box plots show the mean intensity of DCP1 per area in btlGal4 (control), btlGal4>UAS-atg1^RI^,UAS-atg8^RI^ and btlGal4>UAS-fzr^RI^. for the analysis of variance between groups, Welch’s ANOVA (*p* = 0.0001) was applied followed by Dunnett’s T3 multiple group comparison test. (alpha set at 0.05. *p < 0.01, **p < 0.001) *n* = 6 individuals per group. Error bars represent SD of means.

Since we detected higher autophagy in the metameres of the larval DTs eliminated during metamorphosis, we examined whether autophagy might precisely participate in this process. To this end, we began by establishing a reference time point for the elimination of the larval DTs in wild-type animals. Using a UAS-*DsRed* fluorescent marker construct under the control of a *btl*Gal4 driver, we were able to monitor *in vivo* the larval DT through the pupal cuticle. The fluorescently marked tubes formed by the larval DTs in the abdomen of wild-type pupae were visible up to 30 h after pupa formation (APF). From that point on, only scattered fluorescent cells arising probably from the branches of the newly formed adult trachea were observed (Figures 3A,B and I).

Conversely, we detected remnants of the larval DTs as late as 70 h APF upon downregulation of autophagy-related genes in *btl*Gal4>UAS*-DsRed*,UAS*-atg1*^*RI*^ and *btl*Gal4*>*UAS*-DsRed*,UAS*-atg8*^*RI*^ (Figures 3C,D and I). In addition, simultaneous downregulation of *Atg1* and *Atg8*, probably by rendering autophagy inhibition more efficient, allowed the detection of DT remnants even later than 90 h APF (Figure 3E,I). In all these cases, lethality was complete and no individual reached adulthood.

### Examining the dual contribution of autophagy and apoptosis

Apoptosis and autophagy play fundamental roles in tissue remodeling during *Drosophila* metamorphosis, although their respective contribution and interaction are highly tissue-specific [18]. Thus, while the two processes are involved in the degradation of the salivary gland and fat body during metamorphosis [12,14], autophagy but not apoptosis is required in the case of midgut cell death at metamorphosis [15]. As these two processes are interconnected [27,28] and we have recently shown that apoptosis also has an important role in larval tracheal disassembly during metamorphosis [29], we examined the interaction between apoptosis and autophagy in this process.

First, we took advantage of knowledge of the role of autophagy in the reduction and shrinkage of the *Drosophila* midgut in metamorphosis [30]. In particular, in this case there is a bifurcation in the autophagy pathway. In this regard, while *Atg7* and *Atg3* are essential for the autophagy response, they are not required for specific cell reduction. Thus, if the larval trachea was to be reduced by the same mechanism it should not be impaired upon downregulation of *Atg7*. However, disassembly of the larval trachea was not *Atg7*-independent. In particular, RNAi-mediated downregulation of *Atg7* in the trachea impaired the elimination of the DT in the range of impaired elimination caused by the individual or combined downregulation of *Atg1* or *Atg8* (Figure 3F,I). Thus, larval tracheal structures were not removed by activation of the same autophagy mechanism operating in the midgut, thereby suggesting that autophagy induces another process for cell elimination – a clear candidate being apoptosis.

Then, for this purpose, we addressed whether autophagy and apoptosis have an additive effect on larval tracheal disassembly. The combined downregulation of these two processes is a good approach to test this notion [14]. To this end, we assessed in our system the effect of inhibiting apoptosis by a construct that downregulates the three pro-apoptotic genes *rpr* (reaper), *hid* (head involution defective), and *grim* (grim), thereafter referred to as miRHG (see Materials and Methods for details). Upon expression of this construct, triggered by the same *btl*Gal4>UAS*-DsRed* driver, 90% of the pupae at 90 h APF showed larval DT remnants (Figure 3G,I), the maximum phenotype in our assay. The same percentage of individuals with larval DT remnants at 90 h APF was also found when we combined the induction of both miRHG and *atg8*^*RI*^ (Figure 3H,I). Thus, at this extreme time point, this interaction assay used in other systems did not allow us to assess any possible additive effect caused by the combined downregulation of apoptosis and autophagy. As an alternative, we examined the tracheal accumulation of DCP1, a reporter of apoptosis activity, in wild-type pupae and in pupae upon downregulation of components of the autophagy pathway at 12 h APF. DCP1 accumulation in the polyploid larval metameres of the trachea was barely detectable upon the combined downregulation of *Atg1* and *Atg8*, while it was detected in the wild type in the same conditions (Figure 3J,K). Consistently, expression of a *fzr*RNAi construct by means of a *btl*Gal4 driver, which partially switches polyploidy to diploidy, and thus reduces autophagy (see above), also impairs apoptosis (Figure 3J,K). Taken together, our results indicate that the disassembly of the larval DT of the trachea by autophagy in metamorphosis is mediated by autophagy-eliciting apoptosis.

### Polyploidy, autophagy, and cell death

Our study reveals a link between polyploidy and autophagy in the tracheal cells of *Drosophila*. The significance of polyploidy has been widely discussed; however, an emerging topic associated with polyploidy refers to changes in gene expression (see, for instance [31–35]). Nevertheless, changes in the transcriptional signature that could be solely attributed to the polyploidy status are difficult to unravel as the cell populations compared do not serve common functional roles and have distinct characteristics in the respective tissue context. In our model system, cells of the tracheal DT share a common identity and provide a context where populations of diploid and endoreplicating cells with various degrees of polyploidy can be compared directly. In this regard, identifying autophagy as a process linked to the state and the degree of ploidy offers a case for the relevance of the latter. However, the direct consequences of elevated autophagy in polyploid cells are still unclear.

In the system here studied we show how a certain autophagic activity provides a link for elimination of polyploid cells. The “prosurvival” or “prodeath” role of autophagy remains unclear [18]. Moreover, the mere concept of cell elimination by autophagy is controversial as cell elimination can be due to autophagy itself or, alternatively, due to one or more distinct processes triggered or enabled by autophagy (see [36] for discussion and nomenclature). This is the case in the elimination of the tracheal cells in metamorphosis, as autophagy appears to elicit apoptosis. Besides, in this case, the levels of autophagic activity appear to be a crucial element to discriminate between the “prosurvival” or “prodeath” role of autophagy, as autophagy levels in the diploid cells in Tr2 are not associated with cell elimination but instead with cell survival. In this regard, we should emphasize that most probably, not all the autophagy in the larval trachea is restricted to its role in eliciting the apoptosis of the polyploid cells at metamorphosis. In particular, large polyploid cells that lack the molecular elements of stress protection [37] could use autophagy as a “prosurvival” mechanism during a developmental stage with high energy demands. Similarly, the lower levels of autophagy detected in the Tr2 cells might have a protective role at metamorphosis, when a hormonal peak drives progenitor cell activation (for a review see [38]).

Diverse observations have been made on the co-occurrence of autophagy, different degrees of ploidy, and cell death (see, for instance, the review by [39]). However, the link between polyploidy and autophagy does not necessarily give rise to cell elimination. Instead, different outcomes may be expected depending on the cell or tissue context. Along these lines, for example, autophagy driven by polyploidy may contribute to different responses to medical treatments [40]. Thus, the unveiled association of polyploidy with distinct levels of autophagy is likely to be a wide mechanism with multiple consequences for physiology and therapy.

## Materials and methods

### Fly strains and genetics

All fly stocks were raised at 25°C on standard flour/agar *Drosophila* media. The Gal4/UAS system [41] was used to drive the expression of transgenes in trachea at 29°C. To follow the trachea *in vivo* during pupariation, for all the indicated genotypes, flies were allowed to lay eggs for 12 h at 25°C, which were then shifted to grow at 29°C. Pupariation timing for all the selected individuals was scored every 2–3 h during the daytime. The following strains were provided by the Bloomington Drosophila Stock Center (BDSC): UAS-*atg1*^*RI*^ (BDSC 44,034), UAS-*atg8*^*RI*^ (BDSC 28,989), UAS-*atg7*^*RI*^ (BDSC 27,707), UAS-*DsRed* (BDSC 8547), UAS-*fzr* (BDSC 91,688), UAS-*yki*-S167A (BDSC 28,818), UAS-*CycE* (BDSC 4781). The UAS-*fzr*^RI^ (v25553) and the UAS-*yki*^RI^ (v104523) were provided by the Vienna Drosophila Recourse Center (VDRC). The *btl*-Gal4 was obtained from the Kyoto *Drosophila* stock center (DGGR 109,128). The *atg14*^d13^/TM6 and 3×-*mCherry-Atg8a* stocks were kindly provided by G. Juhász (Institute of Genetics, Biological Research Centre, Hungarian Academy of Sciences, H-6726 Szeged, Hungary) [20]. The UAS-mi*RHG* (synthetic microRNA against *reaper*, *hid* and *grim*, is described in [42]). The progeny of *btl*-Gal4 flies crossed to a wild-type strain of a *yw* background was used as a control.

### Immunohistochemistry

For fluorescence imaging, tracheas from L3 wandering larvae and 12-h pupae were dissected in 1X phosphate-buffered saline (PBS; Fisher Bioreagents, BP661–10) and fixed by immersion in 4% formaldehyde (from 37% stock solution [Sigma Aldrich, F8775] in 1X PBS) for 20 and 30 min respectively, at room temperature (RT). The tissues were washed in PBS, blocked in PBT (0.1% Triton X-100 [Sigma Aldrich, T8787], 0.5% BSA [NZYTech, MB04602] in 1X PBS) for at least 1 h and incubated overnight at 4°C with primary antibodies diluted in the same solution. After the incubation with the primary antibodies, the samples were rinsed with PT solution (0.1% Triton X-100 in 1X PBS) (3 × 10 min washes) and incubated with the corresponding secondary antibodies (Alexa Conjugated dyes 488, 555 [Life Technologies, A21206, A31572] 1:500 in PT) for 2 h at RT in the dark. After incubation with the secondary antibodies, the samples were washed in PT (3 × 10 min) and rinsed with PBS before mounting. No antibody incubation was performed for tracheas dissected from the 3×-mCherry-Atg8a larvae, that were mounted for microscopy observation directly after fixation. The following primary antibodies were used: anti-DCP1 (Cell Signaling Technology, Asp216 9578, [1:100]), anti-ref(2)P/SQSTM1/p62 (kindly provided by G. Juhász [23]) [1:1000]), anti-Atg8a (Sigma Aldrich, ZRB 1585, [1:2000]). The tissues were mounted in Vectashield medium with DAPI (Vector Laboratories, H1200). The Tr2 to Tr9 metameres are used for imaging since the anterior (Tr1) and posterior (Tr10) ends of the larval DT are in most cases unavoidably destroyed during dissections.

### RNA extraction and qRT-PCR

After dissection in 1X PBS and isolation of the Tr2 and Tr6 metameres, total RNA was extracted from 6 individuals using the Trizol reagent (Invitrogen, 15596–018), further purified using RNeasy columns (Qiagen, 74104). Reverse transcription was done using the High-Capacity cDNA Archive Kit (Applied Biosystems, 10400745). The 480 LightCycler (Roche) was used for real time PCR reactions. Relative gene expression was normalized to *Act42A* with Tr2 cell populations set to have an expression of one. qRT-PCR experiments were performed in triplicate.

The primer sequences used were:

*Atg8a – F*: 5’- GGTCAGTTCTACTTCCTCATTCG- 3’

*Atg8a – R*: 5’- GATGTTCCTGGTACAGGGAGC- 3’

*ref(2)P – F*: 5’ - GTAAGGACCTTCTGGATC −3’

*ref(2)P – R*: 5’- GTGCATATTGCTCTCGCAC −3’

*Actin42A – F*: 5’-GCGTCGGTCAATTCAATCTT-3’

*Actin42A – R*: 5’-AAGCTGCAACCTCTTCGTCA-3’

### Imaging acquisition, and analysis

Images of dissected tracheas were obtained with the Spectral Leica SP5, Zeiss 780 and Zeiss 880 confocal microscopes, using the HCX PL APO 40×/1,25–0,75 Oil CS and Plan-Apochromat 63×/1.4 Oil DIC M27 objectives. For image acquisition, XY was set to 1024 × 1024and the Z path was set to optimal as defined by the software of each system. The same imaging acquisition parameters were used for all the comparative analyses. Images of pupal tracheas *in vivo* were taken using Macroscope Olympus Fluo MVX10, with Olympus MV PLAPO 1,6× objective. XY was set to 1024 × 1067and the Z path was adjusted to each sample. Images were analyzed using the Fiji software [43]. For stitched image reconstructions, the plugin described in [44] was used. For the quantification of mCh-Atg8a puncta, max intensity projections were used from each individual metamere and a freehand selected area was threshold adjusted to allow segmentation of puncta. The find maxima tool with a prominence of 3 was used to determine the number of the puncta in the defined area. For the quantification of antibody stained ref(2)P and Atg8a puncta in each metamere, max intensity projections were created from 15 slices of Fast Airyscan Acquisitions of the Zeiss 880 system (Airyscan processed by the Zen Lite Software). A defined area was randomly selected within the projections and the subtract background tool with a rolling ball radius of 30 pixels was used before image thresholding for the creation of binary images of the selected areas. For the mCh-Atg8a and ref(2)P quantifications, 5–10 individuals were analyzed for each genotype. The direct estimation of the DNA content for each cell was performed using the DAPI reagent that binds stoichiometrically to DNA [45]. In sum intensity projections that included the whole nucleus of selected DT cells, the intensity of the DAPI channel was measured within the nuclear area and normalized to the mean background intensity in empty areas of the same projections. For the estimation of the C Values, the intensities were normalized to the ones of diploid nuclei of imaginal disc cells from the same preparations that were assigned as 2C. For DCP1 quantifications, sum intensity projections were created from same thickness stacks amongst genotypes and the calculated intensity values for the respective channel were normalized to selected areas corresponding to the Tr6 metamere at 12 h APF. For tracheal remnants scoring in pupae, we included individuals from each genotype in which we could clearly assess visually the presence of DT larval parts within the abdomen of the developing pupae. Final Figures presented in this paper were produced in Inkscape and Adobe Photoshop CC software.

### Statistical analysis

Statistical analysis, data processing and graphical representations were performed in GraphPad Prism 8.4.3 Software. Welch’s two tailed t-tests were used to determine significant differences between two groups to correct for unequal sample distribution variance. For the comparison of more than two groups, Welch’s ANOVA was used, followed by Dunnett’s T3 post hoc tests for multiple comparisons. Statistical significance level α was set at 0.05.

## Supplementary Material

Supplemental Material
