# Supplementary material for: Polyploidy-associated autophagy promotes larval tracheal histolysis at Drosophila metamorphosis
Source: Autophagy. 2023 Jul 9;19(11):2972–81. doi: 10.1080/15548627.2023.2231828 (PMC10549192; doi:10.1080/15548627.2023.2231828)
Supplement: Supplemental Material [file KAUP_A_2231828_SM1764.docx]

**Figure S1.** Differential autophagy along the dorsal trunk of the larval trachea revealed by Atg8a antibody staining. (**A**) Magnification of metameres Tr2-Tr9 from L3 larvae labelled by Atg8a (upper panels in magenta, lower panels white single channel). Cell nuclei are labelled with DAPI, (upper panels in white). Scale bars: 10 μm. (**A ́**) Box plots show the distribution of the values of Atg8a puncta per area in each metamere, which show significant differences between cells of Tr2 and those of the rest of the metameres. For the analysis of variance between groups, Welch’s ANOVA (*p* = 0.0001) was applied followed by Dunnett’s T3 multiple group comparison test. (alpha set at 0.05. **p*<0.05*, **p*<0.005*, ***p*<0.001, *****p*<0.0001). n = 5 individuals per group. Error bars represent SD of means.

**Figure S2.** Autophagy in the trachea depends on the activity of *Atg* genes. (**A**) Representative images of Tr2 and Tr9 metameres of *btl*Gal4 (control), *atg14*^-/-^ and *btl*Gal4*>*UAS*-atg1^RI^* L3 larvae. Magnified views show the increase in the number and size of ref(2)P puncta (in white) in *atg14*^-/-^ and in *btl*Gal4*>*UAS*-atg1^RI^* compared to the control. Scale bars: 20 μm. (**B**) Box plots of ref(2)P puncta per area (upper chart) and average size of ref(2)P puncta (lower chart) along the DT for the same genotypes. For the analysis of variance between groups, Welch’s ANOVA (*p =* 0.0001) was applied followed by Dunnett’s T3 multiple group comparison test, for each metamere. (alpha set at 0.05. **p<*0.05, ***p<*0.005, ****p<*0.0005, *****p<*0.0001). Only statistically significant differences are shown on the charts. n = 6 individuals per group. Error bars represent SD of means.

**Figure S3.** Correlation between polyploidy and autophagy. (**A**) Magnification of Tr6 metameres from L3 larvae of *btl*Gal4 (control), *btl*Gal4*>*UAS-*yki^RI^, btl*Gal4*>*UAS-*fzr^RI^, btl*Gal4*>*UAS-*atg1^RI^,*UAS-*atg8^RI^* and *btl*Gal4*>*UAS-*CycE*. Atg8a, in magenta; cell nuclei are labelled with DAPI, in white. Thermal scale pseudo color represents pixel intensities in sum projections for the DAPI channel. Scale bars: 10 μm. (**A´**) Box plots for mean values of Atg8a puncta/area (left chart) and the summary of C values (right chart) in Tr3-Tr9 of *btl*Gal4 (control), *btl*Gal4*>*UAS-*yki^RI^, btl*Gal4*>*UAS-*fzr^RI^, btl*Gal4*>*UAS-*atg1^RI^,*UAS-*atg8^RI^* and *btl*Gal4*>*UAS-*CycE.* Reduction of polyploidy induced by *yki^RI^* and conversion of polyploid cells to diploid by *fzr^RI^* result in reduced autophagy levels. An increase in polyploidy induced by *CycE* overexpression, leads to increased levels of autophagy. Polyploidy is reduced when autophagy is inhibited by double *atg1^RI^,atg8^RI^*. For the analysis of variance between groups, Welch’s ANOVA (*p =* 0.0001) was applied followed by Dunnett’s T3 multiple group comparison test, for each metamere. (alpha set at 0.05. **p<*0.05, ***p<*0.01, ****p<*0.0005, ****p<*0.0001) n= 6 individuals per group. Error bars represent SD of means.
